# Expanding the phenotypic spectrum of *KCNK4*: From syndromic neurodevelopmental disorder to rolandic epilepsy

**DOI:** 10.3389/fnmol.2022.1081097

**Published:** 2023-01-05

**Authors:** Hong-Jun Yan, Yun-yan He, Liang Jin, Qiang Guo, Jing-Hua Zhou, Sheng Luo

**Affiliations:** ^1^Epilepsy Center, Guangdong Brain Hospital, Guangzhou, China; ^2^Institute of Neuroscience, Key Laboratory of Neurogenetics and Channelopathies of Guangdong Province and the Ministry of Education of China, the Second Affiliated Hospital, Guangzhou Medical University, Guangzhou, China; ^3^Department of Neurology, Women and Children’s Hospital Affiliated to Qingdao University, Qingdao, China; ^4^Department of Neurology, the Affiliated Nanhua Hospital, Hengyang Medical School, University of South China, Hengyang, China

**Keywords:** *KCNK4*, FHEIG syndrome, rolandic epilepsy, molecular sub-regional effects, phenotypic variations

## Abstract

The *KCNK4* gene, predominantly distributed in neurons, plays an essential role in controlling the resting membrane potential and regulating cellular excitability. Previously, only two variants were identified to be associated with human disease, facial dysmorphism, hypertrichosis, epilepsy, intellectual/developmental delay, and gingival overgrowth (FHEIG) syndrome. In this study, we performed trio-based whole exon sequencing (WES) in a cohort of patients with epilepsy. Two *de novo* likely pathogenic variants were identified in two unrelated cases with heterogeneous phenotypes, including one with Rolandic epilepsy and one with the FHEIG syndrome. The two variants were predicted to be damaged by the majority of *in silico* algorithms. These variants showed no allele frequencies in controls and presented statistically higher frequencies in the case cohort than that in controls. The FHEIG syndrome-related variants were all located in the region with vital functions in stabilizing the conductive conformation, while the Rolandic epilepsy-related variant was distributed in the area with less impact on the conductive conformation. This study expanded the genetic and phenotypic spectrum of *KCNK4*. Phenotypic variations of *KCNK4* are potentially associated with the molecular sub-regional effects. Carbamazepine/oxcarbazepine and valproate may be effective antiepileptic drugs for patients with *KCNK4* variants.

## 1. Introduction

The *KCNK4* gene (OMIM* 605720), located at chromosomal locus 11q13.1, is highly expressed in the brain, predominantly in the cerebral cortex and hippocampus. It encodes the potassium two pore domain channels subfamily K member 4 (K2P4.1), a lipid-and mechano-sensitive K+ ion channel with 393 amino acids. The K2P4.1 channel, mainly distributed in the membrane of neurons, plays an essential role in controlling the resting membrane potential and regulating cellular excitability (Brohawn et al., 2012). Previously, only two *KCNK4* variants were identified to be associated with human disease (Bauer et al., 2018; Mariani et al., 2021). The association between *KCNK4* and human disease remains to be further researched with more cases.

Ion channelopathies shared overlapping phenotypes in genes encoding similar ion subunits(Wei et al., 2017). The *KCNK4* gene was previously reported to be associated with facial dysmorphism, hypertrichosis, epilepsy, intellectual/developmental delay, and gingival overgrowth syndrome (FHEIG, OMIM# 618381) (Bauer et al., 2018). It is a syndromic neurodevelopmental disorder that shared notable phenotypic overlaps with the syndrome caused by variants of potassium channel-encoding genes *KCNN3* and *KCNH1* (Gripp et al., 2021). With increasing variants identified, the phenotypic spectrums of *KCNN3* and *KCNH1* are expanding, from syndromic neurodevelopmental disorder to pure epilepsy (Leu et al., 2020; Gripp et al., 2021). There remains room to explore the phenotypic spectrum of *KCNK4.*

In this study, we performed trio-based WES in a cohort of patients with epilepsy without acquired causes. Two novel likely pathogenic *KCNK4* variants were identified in two unrelated cases, including one with Rolandic epilepsy and one with the FHEIG syndrome. The molecular structure alteration was analyzed by 3D protein modeling. We further reviewed all *KCNK4* variants and analyzed the molecular sub-regional effects. The present study expanded the phenotypic and genetic spectrum of *KCNK4*.

## 2. Materials and methods

### 2.1. Subjects

A total of 156 children with epilepsy without any acquired causes were recruited from Guangdong 999 Brain Hospital from June 2020 to March 2021. Clinical information of the affected individuals was collected, including age at onset, type, and frequency of seizures, family history, systemic and neurological findings, and effective antiepileptic drugs. The structural abnormalities were detected by brain magnetic resonance imaging (MRI) scans. Long-term video electroencephalography (EEG) monitoring records were performed with electrodes being arranged according to the international standard of the 10–20 reduced montage system. The EEG results were reviewed by at least two qualified electroencephalographers.

This study adhered to the principles of the International Committee of Medical Journal Editors concerning patient consent for research or participation and received approval from the ethics committee of the Guangdong 999 Brain Hospital. Written informed consent was provided by the patient’s legal guardians.

### 2.2. Whole exon sequencing

Blood samples of the probands and their parents were collected for identifying the source of variants. Genomic DNAs were extracted from blood samples using the Qiagen Flexi Gene DNA kit (Qiagen, Hilden, Germany). WES was performed using a NextSeq500 sequencing instrument (Illumina, San Diego, California, United States) following previously described standard procedures (Wang et al., 2018; Luo et al., 2022). The sequencing data were generated by massively parallel sequencing with an average depth of >125x and >98% coverage of the capture region on the chip for obtaining high-quality reads that were mapped to the Genome Reference Consortium Human genome build 37 by Burrows-Wheeler alignment. Single-nucleotide point variants and indels were called with the Genome Analysis Toolkit.

### 2.3. Bioinformatic analyses

Protein modeling was performed by using the Iterative Threading ASSEmbly Refinement software (I-TASSER^1^; Yang and Zhang, 2015). The confidence of each model was quantitatively measured by a C-score in the range of [−5, 2]. PyMOL Molecular Graphics System (Version 2.3.2; Schrödinger, LLC; New York, United States) was used for three-dimensional protein structure visualization and analysis. The consequences of all the missense variants were predicted by 21 *in silico* tools, including CADD, GERP, SIFT, Fathmm-MKL, fitCons, LRT, M_CAP, polyphen2_HDIV, PolyPhen2_HVAR, MutationTaster, MetaSVM, MetaLR, GenoCanyon, VEST3, Eigen, ClinPred, REVEL, phastCons, phyloP, SiPhy, and PROVEAN. The scores of these tools were obtained from the VarCards database (Li et al., 2018). The Varsite web server was used to analyze the changes in amino acid and hydrophobicity^2^ (Laskowski et al., 2020). The pathogenicity of variants was evaluated by the American College of Medical Genetics and Genomics guideline (ACMG) criterion (Richards et al., 2015).

### 2.4. Statistical analysis

R (version 4.0.3) was used for data processing. The two-sided Fisher exact test was used in the aggregate frequency analysis, which was recommended by ClinGen to compare the frequencies of the *KCNK4* variants between the case-cohort and controls (Consortium, 2015). *p* < 0.05 was considered statistically significant.

## 3. Results

### 3.1. Identification of *KCNK4* variants

**Figure 1:**
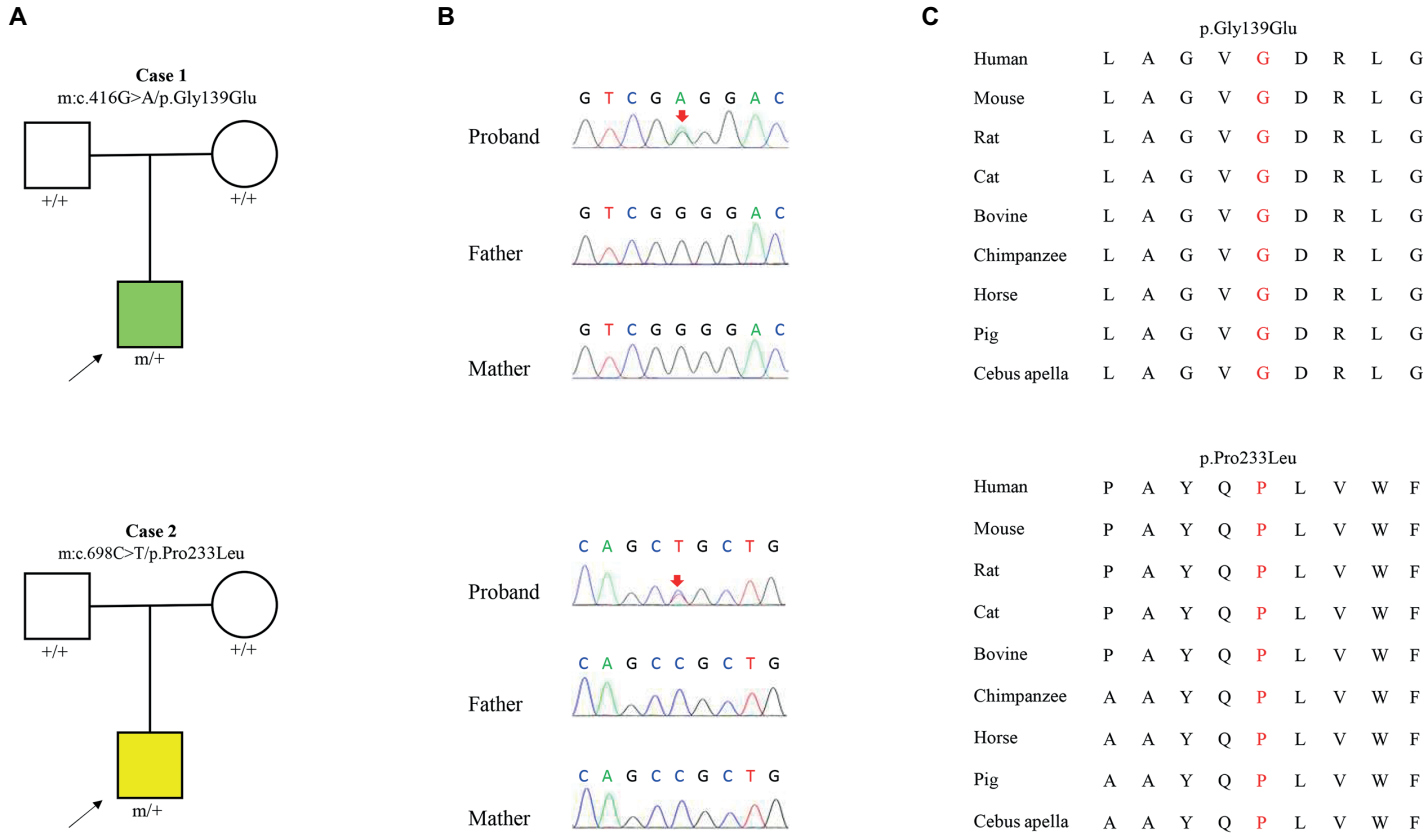
Genetic data of cases with *KCNK4* variants. **(A)** Pedigrees of the two cases with *KCNK4* variants. **(B)** DNA sequence chromatogram of the *KCNK4* variants. Arrows indicate the positions of the mutations. **(C)** The amino acid sequence alignment of the six missense mutations shows that the two variants are highly conserved across species.

Two heterozygous missense variants (c.416G > A/ p.Gly139Glu and c.698C > T/p.Pro233Leu) in the *KCNK4* gene were identified in two unrelated patients, including one with FHEIG syndrome and one with Rolandic epilepsy (Figures 1A,B; Table 1). The two variants were *de novo*, consistent with an autosomal dominant inheritance pattern.

**Table 1:** Clinical features of individuals with *KCNK4* variants.

Source	This study	This study	Ref: (Bauer et al., 2018)	Ref: (Bauer et al., 2018)	Ref: (Bauer et al., 2018)	Ref: (Mariani et al., 2021)
Cases	Case 1	Case 2	Case 3	Case 4	Case 5	Case 6
Variants	c.416G > A p.Gly139Glu	c.698C > T p.Pro233Leu	c.515C > A p.Ala172Glu	c.515C > A p.Ala172Glu	c.730G > C p.Ala244Pro	c.730G > C p.Ala244Pro
Sex	M	M	M	F	M	M
Age	5 yr 4 mo	10 yr	11 mo	8 yr	5 yr 7 mo	6 yr
Inheritance	*de novo*	*de novo*	*de novo*	*de novo*	*de novo*	*de novo*
Seizure timing	Nocturnal and diurnal	Nocturnal mostly	−	NA	NA	NA
Seizure onset	3 yr	1.5 mo	−	<2 yr	10 mo	10 mo
Seizure course	sGTCS 1–3 times/mo for 2 yr	sGTCS 1–3 times/yr for 5 yr	−	GTCS, Ab	sGTCS, 2 times/d	sGTCS
Seizure-free duration	2 yr	2 yr	−	NA	2 yr 6 mo	NA
AEDs	VPA	OXC, VPA	−	LEV, OXC, CPZ, CNZ, Pyridoxine	CPZ	CPZ
EEG	Diffuse slow waves; focal discharges in the left anterior head.	Focal discharges in the left Rolandic region.	Diffuse slow waves	NA	Focal discharge in left parietal, temporal, and occipital regions.	NA
Brain MRI	Normal	Normal	Thin corpus callosum and VVLL enlargement	NA	Mild enlargement of bilateral frontal-insular and temporal subarachnoid spaces	A small-sized adenohypophyseal gland
Development	GDD, severe ID	Normal	GDD, severe ID	GDD, severe ID	Mild speech delay, mild ID	Mild speech delay
Facial dysmorphism	+	−	+	+	+	+
Generalized Hypertrichosis	+	−	+	+	+	+
Gingival overgrowth	+	−	+	+	+	+
Thick hair	+	−	+	+	+	+
Neurological Features	Hypotonia, Hyperreflexia, nystagmus with bilateral optic hypoplasia	−	Hypotonia, Hyperreflexia, nystagmus with bilateral optic hypoplasia	Hypotonia, Intention tremor, nystagmus with bilateral optic hypoplasia	−	NA
Diagnosis	FHEIG syndrome	Rolandic Epilepsy	FHEIG syndrome	FHEIG syndrome	FHEIG syndrome	FHEIG syndrome

Ab, absence seizure; AED, anti-epileptic therapy; CBZ, carbamazepine; CZP, clonazepam; EEG, electroencephalography; F, female; FCS, focal clonic seizure; FHEIG syndrome, facial dysmorphism, hypertrichosis, epilepsy, intellectual disability/developmental delay, and gingival overgrowth syndrome; GDD, global developmental delay; GTCS, generalized tonic–clonic Seizure; ID, intellectual delay; LEV, levetiracetam; mo, months; M, male; MRI, magnetic resonance imaging; NA, not applicable; OXC, oxcarbazepine; VPA, valproic acid; yr, years.

The two variant was not presented in any controls, including the population in the gnomAD database, the ExAC database, the 1000 genome database, and the Epi25 WES Brown database (Table 2). The aggregate frequency of *KCNK4* variants identified in this cohort was statistically higher than that in controls, including the gnomAD-all populations (2/312 vs. 0/282912, *p* = 1.21 × 10^−6^), the gnomAD-East Asian (2/312 vs. 0/19954, *p* = 2.36 × 10^−4^), the ExAC Population (2/312 vs. 0/121416, *p* = 6.55 × 10^−6^), the ESP 6500 Population (2/312 vs. 0/13006, *p* = 5.47 × 10^−4^), the 1000 Genomes Population (2/312 vs. 0/5008, *p* = 3.43 × 10^−3^), and the Epi25 WES Brower Population (2/312 vs. 0/35068, *p* = 7.752 × 10^−5^).

**Table 2:** Aggregate analysis of the frequency of *KCNK4* variants identified in this study.

Identified *KCNK4* variants	Allele count/number in this study	Allele count/number in controls					
		gnomAD-all populations	gnomAD-east Asian population	ExAC population	ESP 6500 population	1000 Genomes population	Epi25 WES brower population
c.416G > A/p.Gly139Glu	1/312	−/−	−/−	−/−	−/−	−/−	−/−
c.698C > T/p.Pro233Leu	1/312	−/−	−/−	−/−	−/−	−/−	−/−
Total	2/312	0/282912	0/19954	0/121416	0/13006	0/5008	0/35068
*p* value*		1.21 × 10^−6^	2.36 × 10^−4^	6.55 × 10^−6^	5.47 × 10^−4^	3.43 × 10^−3^	7.75 × 10^−5^
*OR*		∞	∞	∞	∞	∞	∞
(95% CI)		(170.69-∞)	(12.0-∞)	(73.31-∞)	(7.84-∞)	(3.02-∞)	(21.16-∞)

*p values and odds ratio were estimated with a 2-sided Fisher’s exact test. CI, confidence interval; gnomAD, genome aggregation database; OR, odds ratio.

The *KCNK4* variants identified in this study were predicted to be “Damaged” by numerous *in silico* tools, (16/17 algorithms for p.Gly139Glu and 12/17 algorithms for p.Pro233Leu) (Table 3). The two variants were also predicted to be “Conserved” by four *in silico* algorithms, including GERP, phastCons, phyloP, and SiPhy. Amino acid sequence alignment suggested that the variants were located in residue with high conservation across diverse species (Figure 1C). According to the ACMG guideline, the two variants were rated as “likely pathogenic” due to *de novo* origin (PS2), absence in controls (PM2), and being predicted to be damaged by multiple *in silico* tools (PP3; Table 3).

**Table 3:** Genetic characteristics and ACMG scoring of the KCNK4 variants identified in this study.

	Case 1	Case 2
cDNA change (NM_033310)	c.416G > A	c.698C > T
Protein change (NP_201567)	p.Gly139Glu	p.Pro233Leu
Source	*De novo*	*De novo*
MAF	-	-
ACMG scoring	PS2 + PM2 + PP3	PS2 + PM2 + PP3
ACMG pathogenicity	Likely pathogenic	Likely pathogenic
SIFT	Damaging (0.001)	Tolerable (0.064)
PP2_Div	Probably damaging (1.0)	Possibly damaging (0.879)
PP2_Var	Probably damaging (0.998)	Possibly damaging (0.606)
LRT	Deleterious (0.000)	Deleterious (0.000)
Mutation-Taster	Disease-causing (1)	Disease-causing (1)
PROVEAN	Deleterious (−6.28)	Deleterious (−5.42)
VEST3	Damaging (0.986)	Damaging (0.878)
M-CAP	Damaging (0.093)	Damaging (0.033)
CADD	Damaging (28.6)	Damaging (34)
Fathmm-MKL	Damaging (0.989)	Damaging (0.957)
Eigen	Damaging (0.928)	Damaging (0.453)
GenoCanyon	Damaging (1.000)	Damaging (1.000)
fitCons	Tolerable (0.598)	Tolerable (0.598)
ClinPred	Pathogenic (0.999)	Pathogenic (0.995)
REVEL	Damaging (0.825)	Tolerable (0.297)
MetaSVM	Damaging (0.391)	Tolerable (−1.105)
MetaLR	Damaging (0.587)	Tolerable (0.057)
GERP	Conserved (5.36)	Conserved (4.62)
phastCons	Conserved (1.000)	Conserved (1.000)
phyloP	Conserved (9.610)	Conserved (6.112)
SiPhy	Conserved (16.586)	Conserved (13.611)

ACMG, American College of Medical Genetics and Genomics; CADD, combined annotation dependent depletion; FATHMM, functional analysis through hidden Markov models; Fathmm-MKL, functional analysis through hidden Markov models–multiple Kernels learning; fitCons, fitness consequences of functional annotation; GERP, genomic evolutionary rate profiling; LRT, A likelihood ratio test; MAF, minor allele frequency from genome aggregation database; M-CAP, mendelian clinically applicable pathogenicity; MetaLR, meta logistic regression; MetaSVM, meta support vector machine; NA, not available; phastCons, phylogenetic analysis with space/time models conservation scoring and identification of conserved elements; phyloP, phylogenetic analysis with space/time models computation of *p*-values for conservation or acceleration, either lineage-specific or across all branches; PP2_Div, polyphen2_HDIV; PP2_Var, polyphen2_HVAR; PROVEAN, protein variation effect analyzer; REVEL, rare exome variant ensemble learner; SIFT, sorting intolerant from tolerant; SiPhy, site-specific PHYlogenetic analysis; VEST3, the Variant Effect Scoring Tool 3.0.

No pathogenic or likely pathogenic variants in other epilepsy-related genes were identified in the two patients (Zhou et al., 2018).

### 3.2. Clinical features of patients with *KCNK4* variants

The detailed clinical information of the patients were summarized in Table 1. The missense variant p.Gly139Glu was identified in a boy with FHEIG syndrome. The first seizure occurred when he was 3 years old, mainly presenting as focal to bilateral tonic–clonic seizure (FBTCS). Thereafter, seizures were attacked 1–3 times per month. Since treatment with valproate (24.5 mg/kg/day) and oxcarbazepine (10 mg/kg/day), he became seizure-free. He also exhibits global developmental delay, microcephalus, hypertrichosis, and gingival overgrowth, which are highly consistent with the FHEIG syndrome. Electroencephalogram (EEG) showed diffuse 4–5 Hz slow waves and spikes-slow waves in the right anterior head.

**Figure 2:**
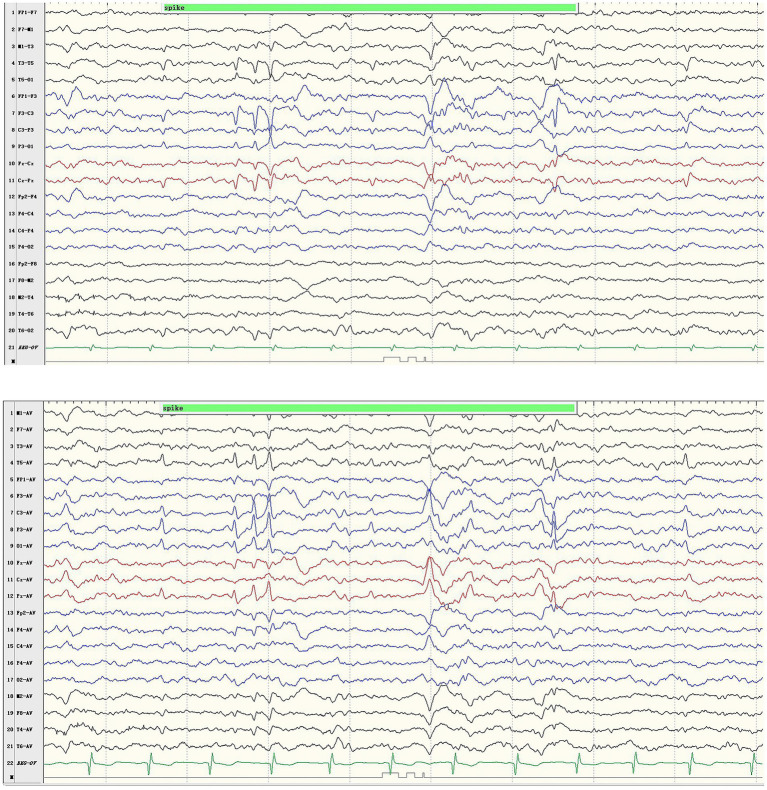
Representative EEG results of case 1. Ictal EEG recorded focal discharges in the centrotemporal area, prominently on the left side.

The boy with the p.Pro233Leu variant presented a mild phenotype of Rolandic epilepsy. He suffered from FBTCS 1–3 times per year since 1.5-year-old. Seizure-free was achieved after being treated with valproate (21 mg/kg/day) and oxcarbazepine (9.1 mg/kg/day). He presented a purely epileptic phenotype without any other phenotype. EEG showed epileptic discharge in the Rolandic area with right-side dominance (Figure 2).

The two patients were born to unrelated parents after an uneventful pregnancy. The brain MRI detected no abnormalities.

### 3.3. Molecular effect of *KCNK4* variants

**Figure 3:**
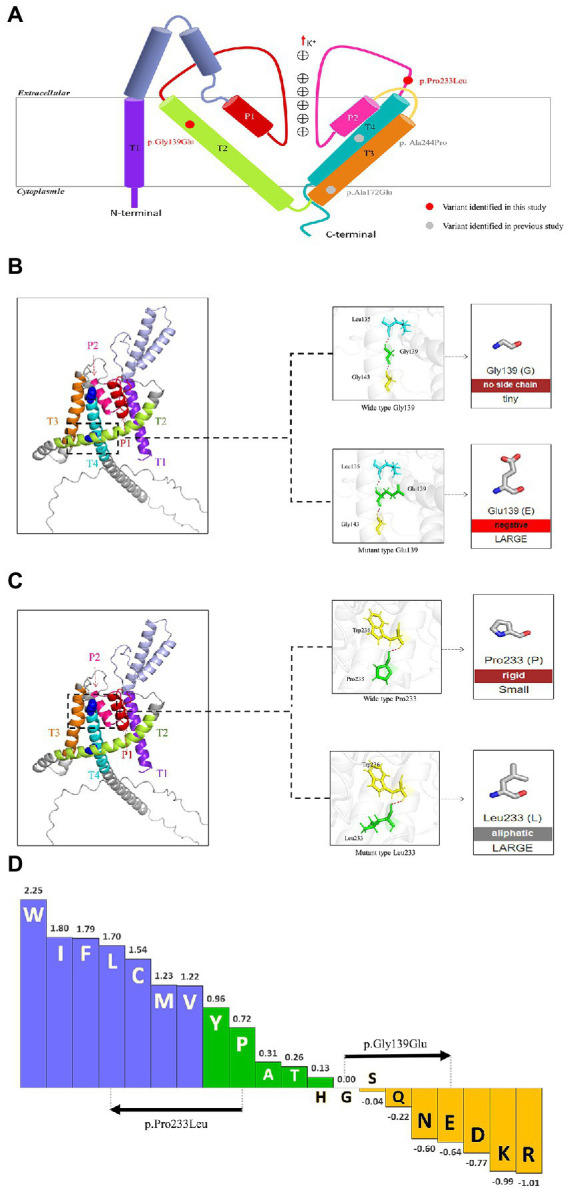
Schematic illustration of *KCNK4* variants **(A)** Schematic of the location of all *KCNK4* variants. Variants identified in this study were shown in red color. Variants reported previously were shown in gray color. **(B)** Protein modeling of the p.Gly139Glu variants. The dashed line box in the left panel indicated the wild-type structure of K2P4.1 channels of p.Gly139Glu. The substitution of Glutamic acid in residue Gly139 was not predicted to alter hydrogen bonds but the polarity of amino acids. **(C)** Protein modeling of the p.Pro233Leu variants. The dashed line box in the left panel indicated the wild-type structure of K2P4.1 channels of the p.Pro233Leu. The substitution of Leucine in residue Pro233 seems not to affect the hydrogen bonds or polarity/charge. **(D)** Fauchère and Pliska hydrophobicity scale exhibited the hydrophobicity of 20 amino acids. Abscissa: from left to right, hydrophobicity gradually decreased. Blue amino acids were hydrophobic, green amino acids were neutral, and yellow amino acids were hydrophilic. Amino acids with high positive values are more hydrophobic, whereas amino acids with low negative values are more hydrophilic.

The *KCNK4* gene encodes a highly conservated lipid-and mechano-sensitive K+ ion channel with 393 amino acids. It contains four transmembrane segments (TM 1–4) and two pore domains (P 1 and P 2; Figure 3A). The variant p.Gly139Glu was located in the TM2 segment, while the variant p.Pro233Leu was located in the linker between the P2 domain and TM4 segment. As shown in protein modeling, the two variants were not predicted to alter any hydrogen bonds forming with the surrounding residues (Figures 3B,C). The substitution of Glutamic acid in residues Gly139 alters the polarity of amino acids (Figure 3B), while the substitution of Leucine in residue Pro233 seems not to affect the polarity/charge (Figure 3C). The two variants are all expected to cause hydrophobicity changes that may lead to functional abnormities, based on the Fauchère and Pliska hydrophobicity scale calculating fifteen physicochemical parameters of the amino acid side chain (Fauchere et al., 1988; Figure 3D).

## 4. Discussions

The *KCNK4* gene encodes the K2P4.1 channel, which is a highly conserved voltage-insensitive potassium channel. The K2P4.1 channel, predominantly distributed in neurons, plays a vital role in controlling the resting membrane potential and subsequently regulating neuronal excitability (Brohawn et al., 2012). To date, only two *KCNK4* mutations were found to be associated with human disease, the FHEIG syndrome. In this study, we identified two *de novo* likely pathogenic variants of *KCNK4* in two unrelated cases. The two patients exhibited distinct clinical phenotypes, including one with Rolandic epilepsy and one with FHEIG syndrome. The variants were novel and not reported previously, with no frequencies in any controls. The aggregative frequencies were significantly higher in the epilepsy cohort than that in the controls. This study suggested that the *KCNK4* gene was potentially associated with epilepsy without other neurological phenotypes. The phenotypic spectrum of *KCNK4* potentially ranged from mild epilepsy such as Rolandic epilepsy to severe syndromic neurodevelopment disorder.

In mice, knockout *Kcnk4* exhibited no abnormalities in the nervous system and no significant susceptibility to pharmacologically induced seizures^3^ (Heurteaux et al., 2004). In the normal control of the gnomAD database, 12 frameshift and 13 stop-gained variants were detected. The probability of being loss-of-function intolerant (pLI) for the *KCNK4* gene is 0.01, indicating that loss-of-function variants of *KCNK4* were not susceptible to natural selection (Karczewski et al., 2020). On the other hand, the pathogenic *KCNK4* mutations reported previously were all missense, which were functionally validated to be gain-of-function (GOF; Bauer et al., 2018). Thus, it is possible that GOF is the pathogenic mechanism and heterozygous missense variants were the main pathogenic genotype for *KCNK4*. The variants identified in this study were all missense, located in highly conserved residues across species, predicted to be damaging by numerous algorithms, and categorized as likely pathogenic by the ACMG criteria. The variants presented limited impact on protein structure (Figure 3), potentially reflecting the less possibility of loss-of-function. Therefore, the two variants are potentially considered to be disease-causing. However, the functional alteration of the two variants remains to be functionally validated.

Previously, two *KCNK4* mutations were reported to be associated with the FHEIG syndrome (Bauer et al., 2018; Mariani et al., 2021). Clinically, the FHEIG syndrome included not only neurological symptoms (epilepsy, development delays, and intellectual disability) but also non-neurological symptoms (facial dysmorphism, hypertrichosis, and gingival overgrowth) (Bauer et al., 2018). The *KCNK4* gene is expressed almost exclusively in the brain^4^. Generally, tissue-specific expression is the basis of gene function and subsequently the clinical phenotype. Such pleiotropy of *KCNK4* is unexpected and low persuasive, and the pure epileptical phenotypes of *KCNK4* are receivable (such as the Rolandic epilepsy in Case 1). Nevertheless, the recurrence of the FHEIG syndrome in Case 2 provided further clinical evidence in supporting the association between *KCNK4* and the FHEIG syndrome. The mechanisms of multisystem symptoms of *KCNK4* are of great challenge and warrant further studies.

Potassium channels play vital roles in neurodevelopmental diseases, particularly in epilepsy. Previously, a series of potassium-encoding genes were initially reported to be the severest phenotypes, such as developmental and epileptic encephalopathy (*KCNA2*, *KCNT1*, *KCNT2*, and *KCNB1*; Barcia et al., 2012; Torkamani et al., 2014; Syrbe et al., 2015; Gururaj et al., 2017) and syndromic neurodevelopment disorder (*KCNN3* and *KCNH1*) (Kortum et al., 2015; Bauer et al., 2019). With increasing identification of variants, these genes were recently found to be associated with mild phenotypes, suggesting the possible phenotypic spectrum for potassium-encoding genes. In this study, we reported the first variant that is associated with Rolandic epilepsy, expanding the phenotypic spectrum of *KCNK4*.

Previous studies suggested that the phenotypic variation was potentially associated with the location of variants, which was known as the molecular sub-regional effect (Tang et al., 2020; Liao et al., 2022). Functional K2P4.1 channels contain four transmembrane segments (TM 1–4) and two pore domains (P 1–2), among which TM4 and TM2-TM3 segment in stabilizing the conductive conformation. Rotation of TM4 about a central hinge seals the intramembrane opening in the conductive state, preventing the lipid block of the cavity and permitting ion entry (Miller and Long, 2012). Additional rotation of TM4 by the TM2-TM3 segment further stabilizes the conductive conformation (Miller and Long, 2012). It is noted that the variants of patients with FHEIG syndrome (case 1 and cases previously reported) are all located in the TM2-TM3 segment or TM4, whereas the variant of the patient with Rolandic epilepsy is distributed in the linker between P2 and TM4 (case 2; Figure 3A). It is speculated that the variants in the area with the function of conductive conformation led to the severe phenotype of FHEIG syndrome, while variants in other areas are possibly associated with mild phenotypes such as Rolandic epilepsy. This potential molecular sub-regional effect may be one of the possible explanations for the phenotypic variation, which needed more cases to confirm.

To date, a total of four *KCNK4* variants (including two from this study) were identified in six patients to be associated with human disease (Table 1). The patients with an identical variant also presented with subtle heterogeneous phenotypes, which might be partially the result of environmental factors and/or modified genes that warrants further investigation. From the perspective of prognosis, almost all patients responded well to carbamazepine/oxcarbazepine and valproate, indicating the potential clinical implication of treatment (Table 1).

In summary, this study expanded the genetic and phenotypic spectrum of *KCNK4*-related disease, providing further clinical evidence in supporting the pathogenic role of *KCNK4*. The phenotypic variation was potentially associated with the molecular sub-regional effect. Carbamazepine/oxcarbazepine and valproate were effective antiepileptic drugs for patients with *KCNK4* variants.

## Data availability statement

The datasets generated for this study can be found in the Genebank repository with the following accession numbers: BankIt2652432 Seq1 OP997444, BankIt2652432 Seq2 OP997445.

## Ethics statement

The studies involving human participants were reviewed and approved by the Ethics Committee of the Guangdong 999 Brain Hospital. Written informed consent to participate in this study was provided by the participants’ legal guardian/next of kin.

## Author contributions

SL and H-JY designed the study and administered the project and revised and write the manuscript with the contribution of all authors. H-JY, QG, and J-HZ completed the collection of the clinical data. Y-yH and LJ analyzed the data.

## Funding

This research was funded by Guangzhou Science and Technology Planning Project (202102080024).

## Conflict of interest

The authors declare that the research was conducted in the absence of any commercial or financial relationships that could be construed as a potential conflict of interest.

## Publisher’s note

All claims expressed in this article are solely those of the authors and do not necessarily represent those of their affiliated organizations, or those of the publisher, the editors and the reviewers. Any product that may be evaluated in this article, or claim that may be made by its manufacturer, is not guaranteed or endorsed by the publisher.

## Footnotes

^1^ https://zhanglab.ccmb.med.umich.edu/I-TASSER/

^2^ https://www.ebi.ac.uk/thornton-srv/databases/VarSite

^3^ http://www.informatics.jax.org/marker/MGI:1298234

^4^ https://www.proteinatlas.org/ENSG00000182450-KCNK4/tissue
